# Factors associated with voice disorders among the elderly: a systematic review^☆^

**DOI:** 10.1016/j.bjorl.2017.11.002

**Published:** 2017-12-26

**Authors:** Amanda Cibelly Brito Gois, Leandro de Araújo Pernambuco, Kenio Costa de Lima

**Affiliations:** a Universidade Federal do Rio Grande do Norte (UFRN), Programa de Pós-graduação em Saúde Coletiva (PPGSCol), Natal, RN, Brazil; b Universidade Federal da Paraíba (UFPB), Departamento de Fonoaudiologia, João Pessoa, PB, Brazil

**Keywords:** Voice disorders, Epidemiology, Cross-sectional studies, Aged, Distúrbios de voz, Epidemiologia, Estudos transversais, Idoso

## Abstract

**Introduction:**

During the aging process, natural modifications occur in the larynx and the structures involved in phonation which explain the specific characteristics found in the voices of elderly persons. When, at any moment, a voice fails and there is interference with communication, a voice disorder has occurred. This can generate disadvantages in communicative efficiency and have a negative impact on quality of life, compromising mechanisms of socialization, the maintenance of autonomy, and the sense of well-being. Nevertheless, there appears to be little clarity about which factors are associated with voice disorders in this population, especially from an epidemiological perspective.

**Objective:**

The present study is a literature review to identify factors associated with voice disorders among the elderly described in population-based studies.

**Methods:**

A systematic review of electronic databases was carried out. The methodological quality of the studies was analyzed with the Strengthening the Reporting of Observational Studies in Epidemiology guidelines. The research was conducted independently by two researchers.

**Results:**

Although two articles met the eligibility criteria, none fulfilled all the criteria for the evaluation of methodological quality. According to the two studies selected for this review, factors associated with voice disorders among the elderly included both physical and psychosocial aspects. However, the methodological discrepancies between the studies, particularly in relation to sample selection and the instruments used indicate great variability and compromise the reliability of the results.

**Conclusion:**

Further prevalence studies and investigations of factors associated with voice disorders in the elderly from an epidemiological perspective, and which involve different cultures, should be carried out.

## Resumo

**Introdução:**

Durante o processo de envelhecimento, modificações naturais ocorrem na laringe e nas estruturas envolvidas na fonação que explicam as características específicas encontradas nas vozes de pessoas idosas. Quando, a qualquer momento, a voz falha e há interferência com a comunicação, ocorre um distúrbio de voz. Isso pode gerar desvantagens na eficiência de comunicação e um impacto negativo sobre a qualidade de vida, comprometendo os mecanismos de socialização, a manutenção da autonomia e o sentido de bem-estar. Entretanto, ainda não estão claros quais os fatores associados aos distúrbios de voz nesta população, especialmente considerando-se uma perspectiva epidemiológica.

**Objetivo:**

O presente estudo é uma revisão da literatura para identificar fatores associados a distúrbios de voz em idosos descritos em estudos de base populacional.

**Método:**

Foi realizada uma revisão sistemática das bases de dados eletrônicas. A qualidade metodológica dos estudos foi analisada utilizando-se as diretrizes *Strengthening the Reporting of Observational Studies in Epidemiology*. A pesquisa foi realizada de forma independente por dois pesquisadores.

**Resultados:**

Embora dois artigos tenham preenchido os critérios de elegibilidade, nenhum deles preencheu todos os critérios para a avaliação da qualidade metodológica. De acordo com os dois estudos selecionados para esta revisão, os fatores associados aos distúrbios de voz em idosos incluíram aspectos físicos e psicossociais. Entretanto, as discrepâncias metodológicas entre os estudos, particularmente em relação à seleção da amostra e aos instrumentos utilizados, indicam grande variabilidade e comprometem a confiabilidade dos resultados.

**Conclusão:**

Devem ser realizados estudos de prevalência e investigações de fatores associados a distúrbios de voz em idosos a partir de uma perspectiva epidemiológica e que levem em consideração diferentes culturas.

## Introduction

The aging process is determined by factors that are present from birth and develop throughout life according to individual variations.^1^ During this process, there are natural changes in the larynx and structures involved in phonation that can explain the specific characteristics found in the voices of elderly persons,^2^ such as hoarseness, breathiness, aphonia, vocal fatigue, effort required to improve vocal projection, a reduction in vocal extension, a tremulous voice, difficulty in controlling vocal intensity, pain in the region of the shoulder girdle, and a sensation of burning, odor or a foreign body in the larynx.^3^

When, at any moment, the voice fails or is perceived by the individual to be different than normal, interfering with communication, a voice disorder (VD) has occurred.^4^ The prevalence of VDs among the elderly is estimated to range from 4.8% to 29.1%^5^ and has a great biological and psychosocial impact,^6^ which can lead to disadvantages in communicative efficiency and have a negative impact on quality of life, compromising mechanisms of socialization, the maintenance of autonomy and the sense of well-being.^7^ In this sense, it is important that caring for the voice of the elderly is based on preventive behaviors and the subsequent increase of vocal efficiency.^8^ To achieve this, it is important to know what factors are associated with VDs among the elderly.

The literature describes how the voices of elderly persons can be affected by the physical, psychic and life history of individuals, as well as bad habits and constitutional, racial, hereditary, alimentary, social and environmental factors.^9^ Despite this, there seems to be little clarity about what factors are associated with VDs in this population, especially from an epidemiological perspective, which can guarantee the representativeness of a given population. Considering this vacuum, the objective of this systematic review of literature was to identify factors associated with VDs in the elderly as described in population-based studies.

## Methods

A systematic review of literature published between January 1900 and October 2016 was performed, including periodicals indexed in the MEDLINE/PubMed, Embase, Scopus, Web of Science, CINAHL, Cochrane, PsycInfo, PAHO, WHOLIS, SciELO and Lilacs/BIREME electronic databases. The search strategy involved the following combinations of search descriptors based on the Medical Subjects Headings (MeSH): (epidemiology) AND (cross-sectional studies) AND (observational studies) AND (associated factors) AND (voice OR voice disorders) AND (aging OR age). The same combinations were used in Portuguese when searching the databases of the SciELO and Lilacs/BIREME electronic databases.

The following inclusion criteria were adopted: original articles published or accepted for publication in English, Spanish or Portuguese, and a population aged 60 and over for developing countries and 65 years of age or older for developed countries, in accordance with World Health Organization criteria. The exclusion criteria applied were articles that considered elderly individuals as part of the sample, but did not explicitly describe the results for this specific group.

The studies were evaluated independently by two different researchers. Afterwards, the analyses were compared and some divergence was observed, which was resolved by consensus. After the articles were identified in the databases, a screening phase was carried out, which consisted of reading the respective titles and abstracts and excluding articles that did not meet the inclusion criteria mentioned above. In the eligibility phase, the remaining articles that had undergone screening and dealt with factors associated with VDs were submitted to a full-text review (Table 1).

**Table 1:** Characteristics of studies included, with methodological quality evaluated in accordance with the STROBE criteria for cross-sectional studies.

Reference	Location	Population	Sample	Gender	Age	Instrument	Factors associated with vocal alterations
Roy et al. (2007)	Utah and Kentucky, USA	Elderly individuals aged 65 years or older	117 elderly individuals	39 (33.3%) men and 78 (66.7%) women	65–94 years (76.1 ± 8.5)	Interview based on adaptation of instrument used in previous study (Roy, Merril, Thibeault, Parsa, Gray & Smith, 2004)	Colds
							Sore throat
							Gastroesophageal reflux
							Arthritis
							Thyroid problems
							Bronchitis
							Sleep disorders
							Feeling anxious/frustrated

Ryu et al. (2015)	Korea	Elderly individuals aged 65 years or older	3759 elderly individuals	1542 (41%) men and 2217 (59%) women	65 years and older (72.4 ± 5.5)	The Korea National Health and Nutrition Examination Survey (KNHANES)	Area of residence
							Body Mass Index (BMI)
							Self-rated health status
							Asthma
							Chronic obstructive pulmonary disease (COPD)
							Thyroid disease
							Cerebrovascular disease
							Vocal fold disease
							Depression

The following data were extracted from the articles that met the eligibility criteria: location of study; sample size; age and gender of participants (minimum, maximum and average); definition of associated factors; diagnostic tools; prevalence and possible biases or comments. The evaluation of the methodological quality of the studies was carried out in accordance with the STROBE criteria.^10^

## Results

**Figure 1:**
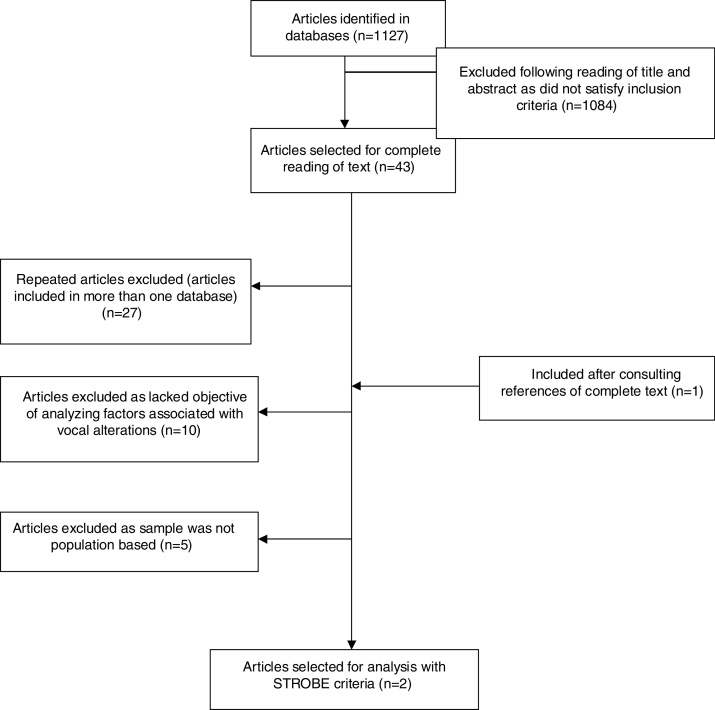
Flowchart for selection of articles.

Among the 1127 initial studies identified by the search strategy, 43 were selected for a review of the full text. Of these, two met the inclusion criteria. Fig. 1 shows the flowchart of the search strategy and Table 1 presents the characteristics of the studies that met the eligibility criteria. The articles included were published in 2007 and 2015 and were conducted in the USA and Korea, respectively.

The two studies exclusively evaluated elderly persons and described distribution based on age and gender. The associated factors investigated by the authors in the articles differed. To obtain their data, the authors of the American article adapted a questionnaire they had generated and used in a previous study carried out with the general population. In the Korean article, data generated from a national survey, entitled the Korea National Health and Nutrition Examination Survey (KNHANES) were used.

In the study conducted in the USA, the factors associated with voice disorders were found to be related to clinical and psychological conditions, such as colds, sore throats, gastroesophageal reflux, arthritis, thyroid problems, bronchitis, sleep disorders and anxiety or frustration. In the Korean study, the associated factors were related to place of residence, Body Mass Index (BMI), self-reported general health status, asthma, chronic obstructive pulmonary disease (COPD), thyroid disease, vocal fold disease, cerebrovascular disease and depression.

Neither of the two studies met all the STROBE criteria (Table 2) following methodological quality assessment. Seven STROBE items (9, 12.4, 12.5, 13.2, 13.3, 16.1 and 16.3) were not covered by either article. In addition, two further items (7 and 11) were only partially achieved, or the data were not clear in both articles. There were methodological discrepancies between the studies. Therefore, it was not possible to perform a meta-analysis.

**Table 2:** Essential items that should be described in observational studies, according to the Strengthening the Reporting of Observational Studies in Epidemiology (STROBE) declaration.

Items	Roy et al. (2007	Ryu et al. (2015)
*Title and abstract*		
1.1 Indicate the study's design with a commonly used term in the title or the abstract	+	+
1.2 Provide in the abstract an informative and balanced summary of what was done and what was found	+	+

*Introduction*		
2. Explain the scientific background and rationale for the investigation being reported	+	+
3. State specific objectives, including any prespecified hypotheses.	+	+

*Methods*		
4. Present key elements of study design early in the paper.	−	+
5 Describe the setting, locations, and relevant dates, including periods of recruitment, exposure, follow-up, and data collection.	+	+
6.1 Give the eligibility criteria, and the sources and methods of selection of participants.	+	+
7. Clearly define all outcomes, exposures, predictors, potential confounders, and effect modifiers. Give diagnostic criteria, if applicable.	?	?
8. For each variable of interest, give sources of data and details of methods of assessment (measurement). Describe comparability of assessment methods if there is more than one group.	?	+
9. Describe any efforts to address potential sources of bias	−	−
10. Explain how the study size was arrived at	?	−
11. Explain how quantitative variables were handled in the analyses. If applicable, describe which groupings were chosen and why.	?	?
12.1 Describe all statistical methods, including those used to control for confounding.	+	+
12.2 Describe any methods used to examine subgroups and interactions	+	−
12.3 Explain how missing data were addressed(“missing data”)	−	−
12.4 If applicable, describe analytical methods taking account of sampling strategy.	−	+
12.5 Describe any sensitivity analyses.	−	−

*Results*		
13.1 Report numbers of individuals at each stage of study; e.g. numbers potentially eligible, examined for eligibility, confirmed eligible, included in the study, completing follow-up, and analyzed	?	−
13.2 Give reasons for non-participation at each stage.	−	−
13.3 Consider use of a flow diagram	−	−
14.1 Give characteristics of study participants (e.g. demographic, clinical, social) and information on exposures and potential confounders.	+	+
14.2 Indicate number of participants with missing data for each variable of interest.	−	−
15. Report numbers of outcome events or summary measures.	+	+
16.1 Give unadjusted estimates and, if applicable, confounder-adjusted estimates and their precision (e.g. 95% Confidence Interval). Make clear which confounders were adjusted for and why they were included.	−	−
16.2 Report category boundaries when continuous variables were categorized.	+	+
16.3 If relevant, consider translating estimates of relative risk into absolute risk for a meaningful time period.	−	−
17. Report other analyses done (e.g. analyses of subgroups and interactions), and sensitivity analyses.	+	−

*Discussion*		
18. Summarize key results with reference to study objectives.	+	+
19. Discuss limitations of the study, taking into account sources of potential bias or imprecision. Discuss both direction and magnitude of any potential bias.	−	+
20. Give a cautious overall interpretation of results considering objectives, limitations, multiplicity of analyses, results from similar studies, and other relevant evidence.	+	+
21. Discuss the generalizability (external validity) of the study results.	+	+

*Other information*		
22. Give the source of funding and the role of the funders for the present study and, if applicable, for the original study on which the present article is based.	−	+

## Discussion

According to the two studies selected for this review, the factors associated with VDs among the elderly included both physical and psychosocial aspects. One associated factor common to both studies was respiratory condition, represented by colds, bronchitis, asthma and COPD. Respiratory diseases are often associated with voice disorders due to inflammatory conditions and edema present in the respiratory mucosa,^11^, ^12^, ^13^ which may affect the structures involved in the physiology of phonation. Another factor that may explain this association would be the side effects produced by the drugs used to combat these conditions, which can affect the salivary glands and respiratory mucus in the elderly.^14^, ^15^, ^16^

Another factor associated with voice disorders was gastroesophageal reflux (GER).^17^ It is proven that digestive system disorders, including GER,^18^ impair the process of phonation by preventing the free movement of the diaphragm, favoring the aspiration of secretions and altering vasomotor functioning by stimulating the vagus nerve.^19^ Physiological changes in the esophagus during aging, such as decreased saliva flow, reduced motility and esophageal sphincter pressure and hiatal hernias may influence the prevalence and severity of GER.^19^ In addition, acid content may harm the larynx and generate inflammation, which diminishes the communicative efficacy of the individual and can cause or aggravate voice disorders.^20^

Thyroid problems were described as factors associated with voice disorders in both studies. Thyroid diseases may be associated with roughness of voice, shortness of breath when speaking, effort when speaking, and uncertainty about how the voice will emerge at the start of emission.^21^ This is because deregulation in the production of thyroid hormones can lead to changes in the lamina propria or excess metabolic processes, which leads to wear of the vocal folds.^22^, ^23^ It can be inferred that regardless of the type of thyroid disease, there may be physiological changes related to the phonatory apparatus, either at vocal or respiratory levels.

VDs among the elderly were also associated with place of residence, BMI and depression.^4^, ^24^ Older people living in urban areas are more exposed to air pollution, which can irritate the pharyngeal and respiratory mucosa and thus trigger some kind of voice disorder.^24^ Body weight, another factor that influences the voice, can affect vocal function through changes in the abdominal respiratory support. When this decreases, the larynx may also undergo physiological and structural changes, including laryngeal muscle atrophy, thinning of the elastic and collagen fibers, and decreased amount of hyaluronic acid in the vocal folds.^25^

Rheumatoid arthritis was mentioned in the American study as a factor associated with VDs. This disease can cause lesions in the larynx, inflammation, edema, swelling and dryness of the vocal folds,^26^, ^27^ with vocal nodules a common finding in these patients.^28^

General state of health can be affected by the physiological modifications in the communication skills during the course of aging^29^ when they begin to interfere in the social life of the elderly.^30^ Alterations in the voice due to changes in the speech apparatus^31^ can have a profound influence on the psychosocial aspects of elderly individuals, interfering in their social functioning.^32^

Vocal fold disease was associated with VDs in the study conducted in the United States. Elderly persons who have voice problems have diseases associated with aging, such as benign vocal fold lesions, inflammatory disorders, laryngeal cancer and laryngeal paralysis.^33^

BMI was also associated with voice disorders in the Korean study. Obese elderly persons may present pathologies such as cardiovascular disease, metabolic syndrome, respiratory diseases such as sleep apnea, psychiatric diseases, neoplasias, dyslipidemias and others,^34^ demonstrating an association with voice disorders, as they can cause physiological changes in the larynx or psychological changes in the individual.

Neurological lesions can cause voice disorders, as in cases of dysarthria, which is the speech disorder caused by neurological injury, which affects motor execution.^35^ Voice disorders caused by brain lesions will not always be specific to the type of lesion, as the recovery of the functions observed in the stable phase of the impairment and the vocal deterioration common to the aging process are often associated.^36^

Sleep disturbance was another factor associated with VDs described in an article. Aging may bring about changes in usual sleep patterns, such as the quantitative reduction of the stages of deep sleep and an increase in the stages of superficial sleep, fragmentation of nocturnal sleep, greater latency in the beginning of sleep and the reduction of the total duration of nocturnal sleep.^37^ These changes may arise from other disorders present in this age group, such as depression, urinary problems and neurological problems, such as Parkinson's disease and strokes,^38^ which are extensively linked to voice disorders.

In terms of psychosocial aspects, it is known that increased anxiety and frustration negatively affect the quality of life of elderly persons.^4^ This association reveals that the voice is a characteristic that reflects the socio-emotional and clinical conditions of the individual, and is considered highly important in human and professional relationships.^39^ Because of this, elderly persons with voice disorders have a higher risk of social isolation, unproductiveness, depression, anxiety and a deterioration of general health status.^40^, ^41^, ^42^

Smoking and alcohol consumption were not associated with voice disorders in either study, which replicates similar findings found in the general population.^12^ The impact of VDs on an individual depends on the characteristics and lifestyle of each person, meaning that such disorders may not be perceived, or may be associated with other factors.^43^ Although this association has been found to be relevant in other studies with specific populations, the voice disorders caused by tobacco or alcohol use may not attract the attention of elderly persons as they are subtle, or the individual may undergo vocal adaptations that mean they do not perceive these changes. Because of this, any voice disorder purely associated with alcohol or tobacco consumption can be so gradual or subtle that it does not worry or attract the attention of the individual.

The form of identification and the recruitment criteria of the selected studies were quite different.^4^, ^39^ One methodological limitation was the extensive instruments that were used to obtain the data, which compromises the reliability of the results, since elderly persons can lose concentration more easily at the moment of the application of the questionnaires.^44^ The use of short, rapid application instruments is suggested, so that elderly individuals present answers with a high degree of reliability and results that are reliable for the reality of this population.

The Korean study^24^ involved the largest population (3759 elderly individuals) and presented physical, behavioral, social and clinical aspects for the definition of associated factors, which made the study more robust.

This review also identified a limitation in terms of the diversity of the locations where the studies were conducted. The articles selected included North American and Asian populations, but no European, African or South American study was found. Factors associated with voice disorders depend on cultural values, lifestyle, socioeconomic-demographic variables, and local climate, among other aspects. Therefore, there is a pressing need to expand the scope of these studies.

According to the results of this review, most voice disorders are associated with physical, social and behavioral health status. These findings may help to develop early screening procedures to identify individuals exposed to these factors and thus propose actions and health services aimed at providing elderly persons with better vocal quality and quality of life during the aging process.

In addition to the previously mentioned limitations, it is worth mentioning that six articles contained samples that were not exclusively composed of elderly individuals, and could have been included if they had explicitly presented the outcome for this population (Fig. 1). The inclusion of these studies could have altered the results. In addition, the studies selected for this review presented methodological bias in relation to sample selection, analysis of results, and the instruments used to collect data, which resulted in heterogeneity between the two.

## Conclusions

This systematic review revealed that factors associated with voice disorders in the elderly included both physical and psychosocial symptoms, although only two studies were found for specific populations, which had differing cultural habits. The methodological discrepancies between the studies, particularly in relation to sample selection and the instruments used imply great variability and compromise the reliability of the results found. It is important to carry out prevalence studies in different cultures investigating the factors associated with voice disorders in the elderly from an epidemiological perspective.

## Ethical approval

This article does not contain any studies with human participants performed by any of the authors.

## Conflicts of interest

The authors declare no conflicts of interest.
